# Unified Strength Model of Asphalt Mixture under Various Loading Modes

**DOI:** 10.3390/ma12060889

**Published:** 2019-03-17

**Authors:** Chengdong Xia, Songtao Lv, Lingyun You, Dong Chen, Yipeng Li, Jianlong Zheng

**Affiliations:** 1 National Engineering Laboratory of Highway Maintenance Technology, Changsha University of Science & Technology, Changsha 410004, China; xiachengdong@stu.csust.edu.cn (C.X.); 15878778765@163.com (D.C.); lypcsust@163.com (Y.L.); zjl@csust.edu.cn (J.Z.); 2 Department of Civil and Environmental Engineering, Michigan Technological University, 1400 Townsend Drive, Houghton, MI 49931-1295, USA; liyou@mtu.edu; 3 Guangxi Communications Design Group Co., Ltd., Nanning 530000, China

**Keywords:** structure design, asphalt mixture, laboratory strength, unified strength model, loading modes

## Abstract

Although the rutting resistance, fatigue cracking, and the resistance to water and frost are important for the asphalt pavement, the strength of asphalt mixture is also an important factor for the asphalt mixture design. The strength of asphalt mixture is directly associated with the overall performance of asphalt mixture. As a top layer material of asphalt pavement, the strength of asphalt mixture plays an indispensable role in the top structural bearing layer. In the present design system, the strength of asphalt pavement is usually achieved via the laboratory tests. The stress states are usually different for the different laboratory approaches. Even at the same stress level, the laboratory strengths of asphalt mixture obtained are significantly different, which leads to misunderstanding of the asphalt mixtures used in asphalt pavement structure design. The arbitrariness of strength determinations affects the effectiveness of the asphalt pavement structure design in civil engineering. Therefore, in order to overcome the design deviation caused by the randomness of the laboratory strength of asphalt mixtures, in this study, the direct tension, indirect tension, and unconfined compression tests were implemented on the specimens under different loading rates. The strength model of asphalt mixture under different loading modes was established. The relationship between the strength ratio and loading rate of direct tension, indirect tension, and unconfined compression tests was adopted separately. Then, one unified strength model of asphalt mixture with different loading modes was established. The preliminary results show that the proposed unified strength model could be applied to improve the accurate degree of laboratory strength. The effectiveness of laboratory-based asphalt pavement structure design can therefore be promoted.

## 1. Introduction

The flexible and rigid pavements are the two most important roads or highways. Over 95% of the roads in the world are flexible asphalt pavements [1,2] because of its good driving comfort [1,3,4], durability [5,6,7,8], and resistance to water damage [9,10,11]. The main material component of asphalt pavement structure is asphalt mixture [12,13]. However, under the dual influence of vehicle load and environmental factors [14,15,16], asphalt pavement will produce different types of diseases. There are three main types of diseases: rutting, low temperature cracking, and fatigue cracking. Rutting is the result of excessive shear deformation due to insufficient shear strength of asphalt mixture, which is related to the high temperature performance of the asphalt mixture [17,18,19,20]. Low temperature cracking is related to low temperature fracture strength of the asphalt mixture [21,22]. Fatigue cracking is mainly related to fatigue strength of the asphalt mixture [8,23]. Especially in recent years, the dramatic increase of heavy-duty vehicles has put forward higher requirements for the structural strength design of asphalt pavement. How to reduce rutting, low temperature cracking, and fatigue cracking of asphalt pavement is an urgent problem to be solved. The structural design of the asphalt pavement belongs to the mechanical–empirical method in China, where the elastic layered half-space is employed to calculate the mechanical response of pavement. The theories of maximum tensile stress and strain are implemented as the failure criterion of asphalt pavement [24,25]. The laboratory strength test methods mainly include direct tension [26,27,28,29], unconfined compression [30,31,32], bending [33,34,35], indirect tension [36,37,38,39], shear [40,41], and triaxial tests [42,43], which are performed to evaluate the tensile, compressive, bending, and shear properties of materials. For the unconfined compression test, the applied strain rate in height direction of specimens is 1.3 mm/(min **^.^** 25 mm) stipulated by AASHTO (American Association of State Highway and Transportation Officials) T 167 and ASTM (American Society for Testing Materials) D 1074. The standard size of Marshall Specimens for indirect tension test is Φ101.6 mm × 63.5 mm, which is adopted in the specifications or standards of United States, Japan, and Australia, while the field-drilling specimen with Φ150 mm is employed in the British standards. Moreover, in these standards, AASHTO T 283, BS EN (British European) 12697-23: 2012, and the Specifications and Test Methods of Asphalt and Asphalts Mixtures for Highway Engineering (JTG E20-2011) used the loading rate of 50 mm/min for the indirect tension test. The unconfined compression test and direct tension test are one-dimensional stress states. The bending test is divided into upper compression and lower tension with the neutral surface as the boundary. The stress at a certain point is the one-dimensional stress state, but the overall stress state is more in line with the stress characteristics of the pavement structure. The center point of the indirect tension test is under vertical compression and horizontal tension, which is in a two-dimensional stress state and conforms to the stress state of the pavement structure. The triaxial test is mainly aimed at the stress characteristics of asphalt pavement under a complex stress state. However, the strengths obtained by different test methods are usually quite discrepant, and it is difficult to compare between them. Therefore, the arbitrariness of the strengths of asphalt mixture under different loading modes should be considered during the asphalt pavement structure design and the related risk management.

The strength of asphalt mixture and other related mechanical parameters have always been a common topic for the civil engineers and researchers. Su et al. [44] used the superpave indirect tensile (IDT) strength test to evaluate the concrete strength of reclaimed asphalt pavement (RAP). It was found that the IDT strength of concrete decreased with the increase of percentage of RAP and temperature. Saride et al. [45] studied the RAP/VA (Reclaimed Asphalt Pavements/Virgin Aggregates) mixture stabilized by alkali activated fly ash. It was found that the strength of the mixture meets the strength requirements of the specification. Ji, X et al. [46] stated that UPT-NSM (Uniaxial Penetration Test—Numerical Simulation Method) can be utilized to optimize the gradation better than the step-filling test to improve the shear strength and rutting resistance of an asphalt mixture. It was noted that the anti-shear strength and dynamic stability of graded asphalt mixture optimized by UPT-NSM are 25.5% and 27.0% higher than the specified gradation, respectively. Li et al. [47] studied the influence of production conditions on the indirect tensile strength characteristics of foamed asphalt mixture. The strength characteristics of the same graded foamed asphalt mixture were mainly affected by curing time, cement dosage, and asphalt content, and it almost had nothing to do with the foam characteristics. Gaus et al. [48] explored the use of button granular asphalt (BGA) instead of petroleum asphalt to produce asphalt concrete bearing course (AC-BC) mixes. Compared with an AC-BC mixture without BGA, the application of BGA partly replacing petroleum asphalt in AC-BC mixture improves its compressive strength and elastic modulus. There was no significant difference in the Poisson ratio of all mixtures. 

At present, numerous factors affecting the strength of asphalt mixtures have been reported by laboratory and field researchers and there were many useful conclusions that were established. However, the strengths that were obtained using different test methods are still tough to compare, which leads to the randomness of the strength indexes in asphalt mixture design. The negative impacts on the design and analysis of asphalt mixtures form these randomness indexes are self-evident. Fortunately, there are many studies have been reported by the researchers for unified parameter model of materials. For example, Yu, M.H et al. [49,50] proposed a unified strength criterion for rock considering the effect of intermediate principal stress. Its strength parameters can be determined using conventional triaxial compression tests. It is found that the unified strength theory can be used to describe various types of rock. You, M et al. [51] put forward that the unified strength theory of linearity and nonlinearity is constructed directly in the form of principal stress. Based on the test results of true triaxial compression, conventional triaxial compression and elongation of rocks, the material parameters and fitting deviations in several strength criteria and their applicability are determined. Danni et al. [52] found that the strength properties of high-strength concrete (HSC) under multi-axial stress may be conducted via shear-type four-parameter unified strength theory (STFP-UST) and nonlinear unified strength theory (N-UST) through analysis of the failure surfaces of several twin shear strength criteria. Wu et al. [53] introduced a shape factor that is expressed as a function of the corner radius ratio, ρ = 2*r*/*b*ρ = 2*r*/*b*. Doing so, a unified model for the concrete strength of FRP-confined columns that have an arbitrary corner radius is described. This model can be degenerated into two special cases for circular columns and sharp cornered square columns when ρ = 1ρ = 1 and 0, respectively. Through collecting all of the available experimental results on both circular and square columns from the open literature for model evaluation, a comprehensive and updated database has been established. A better correlation of the proposed model has been demonstrated by comparing between the test results and the model predictions. Wu, Y.F et al. [54] also proposed a new model based on the Hoek–Brown failure criterion. The existing strength models for FRP (Fiber-Reinforced Polymer)-confined circular and square concrete columns are reviewed, evaluated, and compared with the proposed model. Then, using an updated database, a large number of test data is to evaluate the models. A comparison between the models and the test results is used to demonstrate the accuracy of the proposed model. In addition, the model has a unified form for both circular and square columns. It can be used to predict the strength of columns that have existing damage or cracks. Wei et al. [55,56] presented a new stress–strain model for FRP-confined concrete columns. One of advantages of the model is its unified form (mathematical expression). Compared with the test results, the model can be predicted the ultimate stress and strain more accurately, particularly the strain. You et al. [57,58] established a three-dimensional (3D) microstructure-based computational model through applying a coupled thermo-viscoelastic, thermo-viscoplastic, and thermo-viscodamage constitutive model. The result reflected that the generated 3D microstructure model and the presented constitutive model could be implemented effectively to predict the overall thermo-mechanical response of asphalt concrete. Hajj et al. [59] proposed a unified permanent deformation model, which uses response measurements of two tests. The new model quantifies the accumulated permanent shear strain as a function of the number of load cycles and factor of safety (FOS). The safety factor is defined in q-p space and evaluated according to the applied stress and triaxial compressive strength characteristics (cohesive force and internal friction angle). For specific mixtures used in this study, there is a good correlation between cumulative permanent shear strain and FOS level regardless of stress conditions and test types.

The above researches laid a foundation for establishing the unified strength model of asphalt mixture. However, the mentioned-above research on the unified strength model of materials mainly applied on cement concrete and rock materials, while the related unified parameter model employed on asphalt mixture is usually based on the computation model revealed the thermos-mechanical response and permanent deformation of asphalt mixture [60,61]. Owing to the complex composition and structure of asphalt mixtures and the various destruction forms, there is little research on the unified strength model of asphalt mixtures.

Therefore, direct tension, unconfined compression, and indirect tension strength tests under different loading rates were carried out in this paper. A unified strength model of asphalt mixture under different loading modes was established by using the relationship between the strength ratio of direct tension, unconfined compression, and indirect tension tests and the loading rate ratio. 

The main objectives of this study are to reveal the strength rate characteristics of asphalt mixtures under various loading modes and establish a unified strength model to solve the uncertainty of the strength parameters of asphalt mixtures under various loading modes. The direct tension, indirect tension, and unconfined compression tests were applied in the study.

## 2. Materials and Sample Preparations

### 2.1. Materials

In this paper, strength tests of direct tension, indirect tensile, and unconfined compression were implemented separately to establish a unified strength model of asphalt mixture under different loading modes. The dense gradation asphalt mixture AC-13C that was composed of SBS (styrene-butadiene-styrene) modified asphalt made of Xiamen Huate group Co., Ltd, Xiamen, China and limestone aggregates produced in Shizichang, Niujiaowu, Foshan City, China was chosen. The performance indexes of SBS modified asphalt are shown in Table 1, the densities of limestone aggregate are shown in Table 2, and the properties of the aggregate are shown in Table 3. 

**Figure 1:**
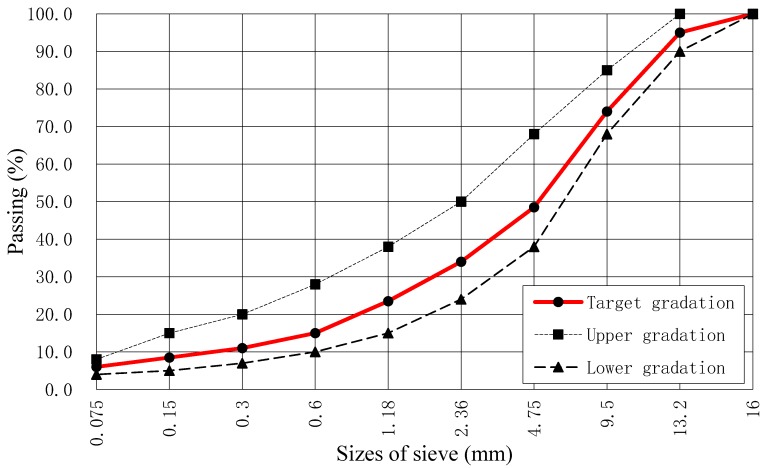
Aggregate gradation curve of dense graded asphalt mixture (AC-13C).

Through the above test results, it was shown that SBS modified asphalt and the aggregate satisfied the requirements of JTG F40-2004 [62], which were the technical specifications for asphalt pavement construction in China. The aggregate gradation curve of dense graded asphalt mixture (AC-13C) and the target gradation of the asphalt mixture is shown Figure 1. The optimum asphalt content was determined using the Marshall tests, and the test results are displayed in Table 4.

### 2.2. Sample Preparations

**Figure 2:**
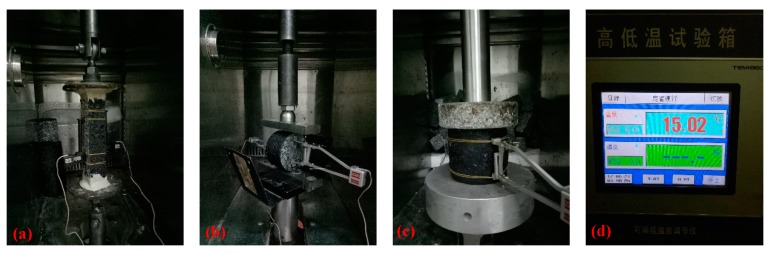
Testing process of strength under different loading modes: (**a**) direct tension test, (**b**) indirect tension test, (**c**) unconfined compression test, and (**d**) the environmental chamber.

According to the Specifications and Test Methods of Asphalt and Asphalts Mixtures for Highway Engineering (JTG E20-2011) [63], the block samples of asphalt mixture plates were made through the method of vibration wheel grinding. Along the rolling direction, each beam was cut to a length, width, and height of 250 mm, 50 mm, and 50 mm, respectively, for direct tension specimens. The cylindrical specimens for the unconfined compressive fatigue test were made using an SGC (Superpave Gyratory Compactor) with a size of Φ100 mm × 100 mm, and the indirect tensile specimens were prepared by slicing the top and bottom surface of the specimens of unconfined compressive moduli test to the size. The cylindrical specimens with height of 100 ± 2mm and diameter of 100 ± 2 mm of asphalt mixture made using a SGC gyratory compactor were prepared for unconfined compression. In addition, the indirect tensile specimens were prepared by slicing the top and bottom surface of the specimens of unconfined compressive moduli test to the height of 60 ± 2 mm and diameter of 100 ± 2 mm. Then, the asphalt mixture specimens were put in the environment chamber at 15 °C for 4–5 h. Subsequently, it was placed on the strength test support of MTS (Material Testing System)-Landmark. The preliminary contact between the indenter of the strength test and the specimen was adjusted to start the test, and the test process was completed in environment chamber. The set-up details of the strength tests are shown in Figure 2.

## 3. Test Results and Analysis

The displacement control mode for China, the United States, and Europe was adopted in the loading rate control mode of strength test. Among them, the loading rate of the unconfined compressive strength test with the size of Φ 100 mm × 100 mm was 2 mm/min and 5.08 mm/min for AASHTO T167 and JTG E20-2011, respectively. The loading rate for indirect tensile strength was 50 mm/min for AASHTO T 283, BS EN 12697-23: 2012, and JTG E20-2011. The loading rate of flexural strength was also 50mm/min for JTG E20-2011. However, the test methods of direct tensile strength were not clearly given in this regulation. In order to explore the loading rate characteristics of the asphalt mixture strength under different loading modes, the displacement loading rate of test regulations could not be unified, so the stress loading rate control mode was adopted, and the test temperature was unified at 15 °C.

### 3.1. Direct Tensile Strength Test at Different Loading Rates

Under the stress control mode, the direct tensile strength tests of asphalt mixtures at different loading rates were carried out, and the results are shown in Table 5.

**Figure 3:**
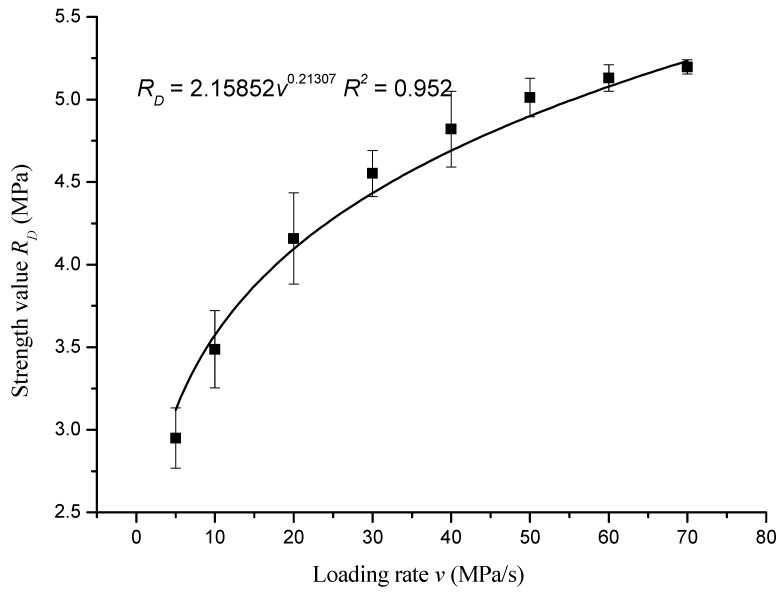
The curve of direct tensile strength of asphalt mixture with loading rate.

The strength values in Table 5 were fitted with the loading rate. The fitted curve is shown in Figure 3.

The fitting equation was as follows:

*R_D_* = 2.15852*v*^0.21307^, *R*^2^ = 0.952 (1)

According to the fitting results, the direct tensile strength *R_D_* of the asphalt mixture varied with the loading rate *v* as a power function. The strength increased with the increase of loading rate, and the rate of strength increase slowed down with the increase of loading rate.

### 3.2. Indirect Tensile Strength Test at Different Loading Rates

According to Chinese Standard Test Methods of Bituminous and Bituminous Mixtures for Highway Engineering (JTG E20-2011) [63], the indirect tensile strength tests of asphalt mixtures under different loading rates were performed. The results of the tests are shown in Table 6.

**Figure 4:**
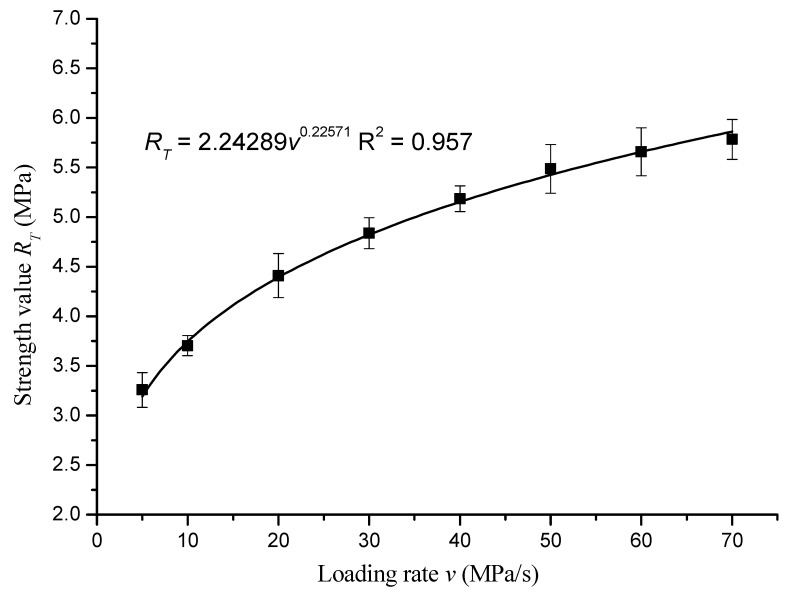
Indirect tensile strength curve of asphalt mixture with loading rate.

The strength values in Table 6 were fitted with the loading rate. The fitted curve is shown in Figure 4.

The fitting equation was as follows:

*R_T_* = 2.24289*v*^0.22571^, *R*^2^ = 0.957 (2)

According to the fitting results, the indirect tensile strength *R_T_* of asphalt mixture varied with the loading rate *v* as a power function. The strength increased with the increase of the loading rate, and the rate of strength increase slowed down with the increase of the loading rate.

### 3.3. Unconfined Compressive Strength Test at Different Loading Rates

Considering the test threshold of employed material testing system (MTS) was 100 kN, through the tentative experiments it was found that the unconfined compression failure load exceeded 100 kN when the loading rate was greater than 3 MPa/s. For the sake of safety and operability of the test, the strength values at the loading rates that exceeded the threshold of MTS were obtained using the delay prediction method in this study. The proposed delay prediction method was performed based on enough laboratory test data within the threshold of MTS, such that the strength values at the loading rate that exceeded the threshold of MTS could be output from the fitting curve. The test results are shown in Table 7.

**Figure 5:**
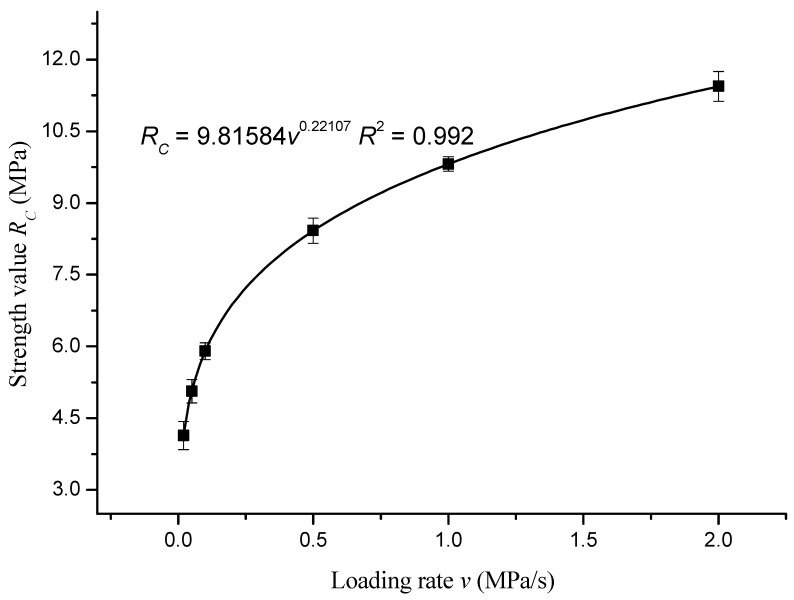
Unconfined compressive strength versus loading rate of asphalt mixture.

The strength values in Table 7 are fitted with the loading rate. The fitted curve is shown in Figure 5.

The fitting equation was as follows:

*R_C_* = 9.81584*v*^0.22107^, *R*_2_ = 0.992 (3)

According to the fitting results (*R_C_* = 9.81584*v*^0.22107^ and *R*_2_ = 0.992), the unconfined compressive strength *R_C_* of the asphalt mixture varied with the loading rate *v* yielding to a power function. The strength values of asphalt mixture at the loading rates of 5 MPa/s, 10 MPa/s, 20 MPa/s, 30 MPa/s, 40 MPa/s, 50 MPa/s, 60 MPa/s, and 70 MPa/s were predicted as 14.01 MPa, 16.33 MPa, 19.035 MPa, 20.82 MPa, 22.187 MPa, 23.309 MPa, 24.267 MPa, and 25.109 MPa, respectively.

### 3.4. Research on Strength Parameters based on Mohr–Coulomb Theory

Asphalt mixture is mainly composed of asphalt and aggregate. The cohesive force is mainly provided by asphalt. The internal friction angle can occur when aggregates are embedded. At present, Mohr–Coulomb theory can be widely used to analyze the strength parameters of asphalt mixtures when the strength characteristics of asphalt mixtures are researched. Based on Mohr–Coulomb theory, cohesive force *C* and internal friction angle *φ* can be obtained using a triaxial test, and a tension and compression test. Triaxial test equipment is complex, expensive, and difficult to operate. Although the real stress state of pavement can be well simulated by it, it has certain limitations to get actual application and project popularization.

It is convenient to determine the cohesive force *C* and internal friction angle *φ* of asphalt mixture through a direct tensile strength test and unconfined compressive strength test. The material and mechanical assumptions are that the material composition variables, the mechanical excitation variables, and the intrinsic parameters of the two tests are the same. After the *R_C_* and *R_D_* obtained from unconfined compressive and direct tensile strength tests, the two parameters can be calculated according to the conversion Equations (6) and (8) given below. The conversion relations can be derived from a Mohr circle.

**Figure 6:**
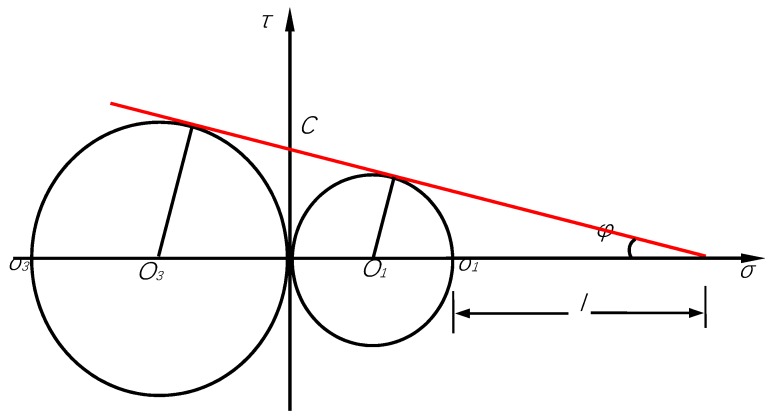
Mohr circle diagram for determining the values of *C* and *φ* through unconfined compressive and direct tensile strength.

When direct tensile test was carried out, *σ*_1_ = *R_t_* and *σ*_3_ = 0; when unconfined compression test was implemented, *σ*_1_ = 0 and *σ*_3_ = −*R_C_*. According to the geometric relationship in Figure 6:

(4) $$\frac{l+\sigma_{1}/2}{l+\sigma_{1}+\left|\sigma_{3}\right|/2}=\frac{\sigma_{1}}{\left|\sigma_{3}\right|}$$

Substituting the above conditions into Equation (4) to obtain:

(5) $$l=\frac{R_{D}^{2}}{R_{C}-R_{D}}$$

In the right triangle:

(6) $$sin\varphi =\frac{\sigma_{1}/2}{l+\sigma_{1}/2}=\frac{R_{D}}{2l+R_{D}}=\frac{R_{C}-R_{D}}{R_{C}+R_{D}}$$

*C* is the intercept between straight line and ordinate. From Equation (6), *tanφ* is calculated as follows:

(7) $$tan\varphi =\frac{R_{C}-R_{D}}{2\sqrt{R_{C}R_{D}}}=\frac{C}{l+R_{D}}$$

The solution *C* is obtained below:

(8) $$C=\frac{\sqrt{R_{C}R_{D}}}{2}$$

The results of unconfined compressive and direct tensile strength tests in Table 5 and Table 7 are substituted for Equations (7) and (8) to calculate the cohesive force *C* and internal friction angle *φ* at different loading rates, as shown in Table 8.

**Figure 7:**
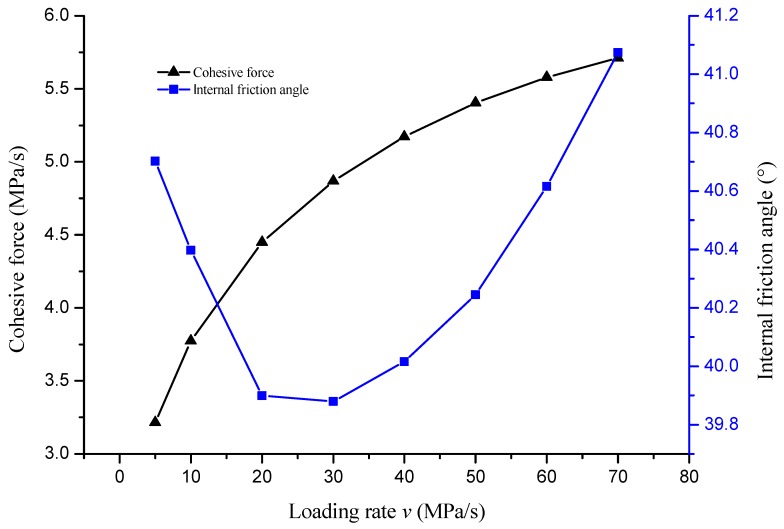
The variation of cohesive force and internal friction angle with loading rate.

The variation of cohesive force and internal friction angle with loading rate is shown in Figure 7.

Figure 7 shows that the cohesion increased sharply with the increase of loading rate, and then the growth rate tended to be gentle. Equation (8) also shows that cohesive force *C* is half of the geometric average value of unconfined compressive strength *R_C_* and direct tensile strength *R_D_*, so the loading pattern of cohesive force was consistent with that of unconfined compressive strength and direct tensile strength. The cohesive force of mixtures was mainly provided by the cementation between asphalt and aggregate. When the loading rate was high, the material exhibited more low-temperature morphology and the cohesive force was greater. The pattern of variation of internal friction angle with loading rate was not as clear as for the cohesive force. The pattern of variation decreased first and then increased in the experiment. The pattern of variation was that the internal friction angle decreased with the loading rate and tended to be flat in theory. The hypothesis of the theoretical pattern of variation was that the material and mechanical parameters of unconfined compression and direct tension were the same. In fact, it was difficult to satisfy the hypothesis under the experimental conditions.

### 3.5. Preliminary Explanation of Strength Discrepancy of Various Loading Modes

Asphalt mixture is usually used as the surface material of asphalt pavement, which directly bears various vehicle loads and environmental factors [64]. It is a kind of composite material, which mainly consists of asphalt, coarse aggregate, fine aggregate, and filler. These materials of different quality and quantity are mixed to form different structures, which have different mechanical properties.

The research on the composition and structure of asphalt mixture mainly includes surface theory and mortar theory. Surface theory holds coarse aggregate, fine aggregate, and filler to form a mineral skeleton. Asphalts binders with bonding ability are distributed on the surface of mineral skeleton and cemented into a whole structure. According to the theory of mortar, the mixture is a kind of dispersed system with a multi-level spatial network cementitious structure. The dispersed system includes a coarse dispersed system (asphalt mixture), subdivided dispersed system (asphalt mortar), and differential dispersed system (asphalt mastic).

The size and distribution of mineral aggregates in asphalt mixture, the position of aggregates, and the ratio of closed voids to connected voids of asphalt mixture are all important parts of its structure. Their differences will have a great impact on the mechanical properties of asphalt mixture. The properties of asphalt mixture improved its structure, especially the interaction between aggregate and cementing material, which made the chemical bond between the two materials and the mixture become a cohesive structure with high strength. Usually, the spatial structure of asphalt mixture is a cementitious structure. In this structure, the main factors determining the anti-destruction performance of asphalt mixture are the cohesive force between aggregates under the action of asphalt mortar, the embedding effect between aggregates, the internal friction resistance between coarse aggregates and fine aggregates, etc.

Under the condition of direct tensile test, asphalt mixture specimens are subjected to tensile stress. The deformation of asphalt mixture makes the aggregate pull apart, and the binder filled between aggregates plays a good bonding role, which is mainly borne by the cohesive force between asphalt and aggregate and the cohesive force of asphalt. The direct tensile strength is the smallest compared with the strength values under the other two test conditions. Under the condition of indirect tensile test, the asphalt mixture is in a bidirectional stress state. The compressive properties depend on the embedding effect of the aggregate, and the transverse tensile properties depend on the cohesive force and internal friction between asphalt mortar and aggregate or asphalt. Under the condition of unconfined compressive strength test, asphalt mixture specimens are subjected to compressive stress. Under the action of compressive stress, the aggregate particles are close to each other, and the skeleton formed by the aggregate particles begins to play a role. The loading is mainly borne by the internal friction resistance and the embedding force formed by the aggregates, so the compressive strength is greater than that under the other two tests. In summary, when the material is in different loading modes, the external factors that determine the anti-destruction performance of materials is inconsistent, which is the main reason for the differences of the tensile, compressive, and indirect tensile strength parameters of the material, and the fundamental reason why the compressive strength is greater than the tensile strength in general.

At the same time, it can be seen from Figure 6 that the growth rates of the strength of direct tension, indirect tension and unconfined compression with the loading rate were varied. Among the three loading modes, the growth rate of the unconfined compressive strength was the largest, followed by the indirect tensile strength, which was related to the stress state and failure mode of the three.

### 3.6. Unification of the Relation Between Strength and Loading Rate under Different Stress Conditions

From Table 6 and Table 7, the relationship between strength and loading rate under various loading modes can be determined. The average strength values of various loading modes at eight different loading rates from 5 MPa/s to 70 MPa/s were compared, as shown in Table 9. It should be noted that the relationship between the loading rate and vehicle speed can be expressed using the equation *v* = *p*/(*l*/*t*), where *v* is the loading rate, *t* is the vehicle speed, *p* is the tire–pavement contact pressure, and *l* is the length of the tire ground. In general, the length (*l*) of the tire ground is 0.1 m, and the tire-pavement contact pressure *(p*) is 0.4 MPa. In this study, the loading rates of 5 MPa/s, 10 MPa/s, 20 MPa/s, 30 MPa/s, 40 MPa/s, 50 MPa/s, 60 MPa/s, and 70 MPa/s were adopted in the strength test of asphalt mixtures. Their corresponding vehicle speeds are 4.5 km/h, 9 km/h, 18 km/h, 27 km/h, 36 km/h, 45 km/h, 54 km/h, and 63 km/h, respectively.

**Figure 8:**
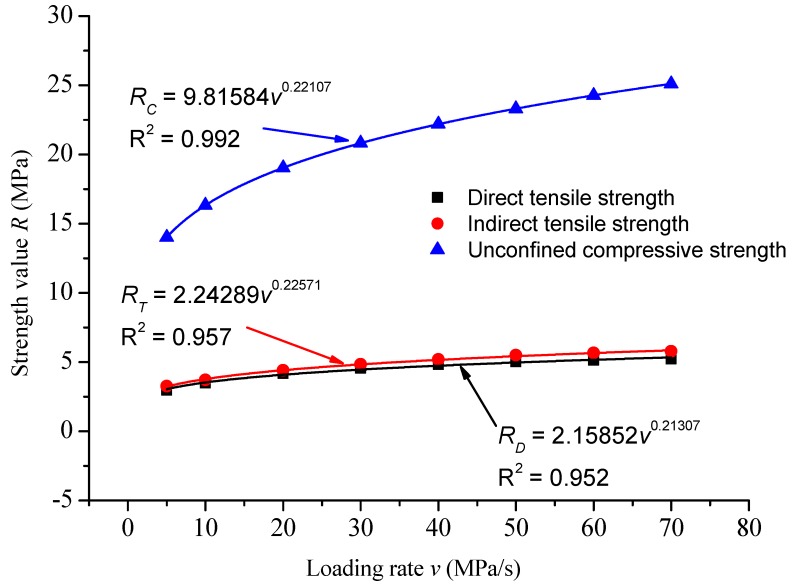
Comparison of the relationship between strength and loading rate under various loading modes.

The relationship between strength and loading rate under various loading modes was compared, as shown in Figure 8.

The fitting curve parameters of the relationship between strength and loading rates are summarized in Table 10.

It can be seen that the strength–loading rate curves of various loading modes increased with the increase of loading rate, and the strength decreased with the increase of loading rate. Under the same loading rate, the direct tensile strength was close to the indirect tensile strength, and the unconfined compressive strength was far greater than the direct tensile strength and the indirect tensile strength. The parameters of the strength–loading rate curves under various loading modes were quite different, which brings a lot of inconvenience to the experimental research. This section will use standardized methods to unify the relationship between strength and loading rate, as shown in Table 11.

**Figure 9:**
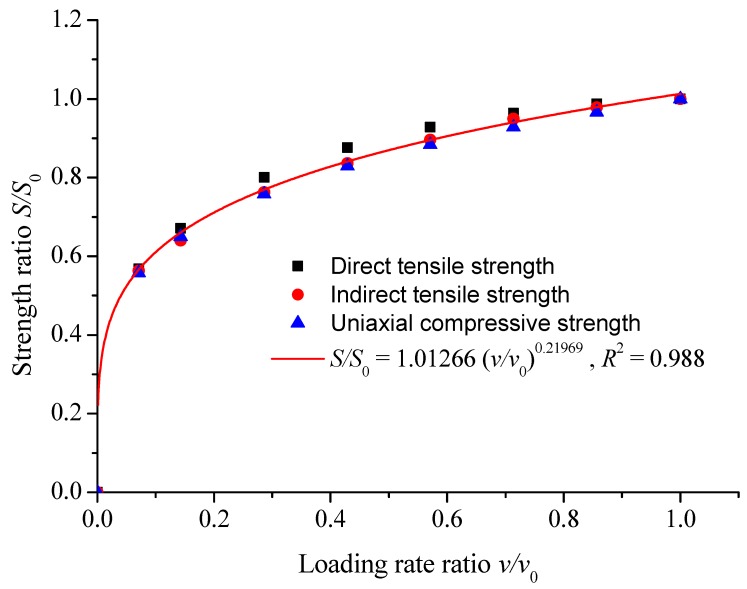
Strength ratio versus loading rate ratio under various loading modes.

The strength ratio and loading rate ratio of direct tension, unconfined compression, and indirect tension in Table 10 are fitted in Figure 9.

The fitting equation was as follows:

*S/S*_0_ = 1.01266 (*v/v*_0_)^0.21969^, *R*^2^ = 0.988 (9)

where, *S* is the strength values of various loading modes at different loading rates, MPa; *S*_0_ is the strength values of various loading modes at 70 MPa/s loading rate, MPa; *v*_0_ is the set loading rate, 70 MPa/s.

As shown in Figure 6, the relationship between strength and loading rate under various loading modes can be obtained by using the traditional power function equation, but the parameters of the equation could not be unified, and the difference was large. As shown in Figure 7, the relationship between strength and loading rate of direct tension, unconfined compression, and indirect tension could be unified by using the equation of strength ratio and loading rate ratio, and the correlation coefficient was better. By standardizing the curve function of strength ratio and loading rate ratio under various loading modes, the strength values of various loading modes under other loading rates could be predicted through the strength values of one stress state, and the strength values of different loading rates under other stress states could be predicted.

## 4. Summary and Conclusions

In this study, considering the main objective of this study was to solve the uncertainty of the strength performance of asphalt mixture under various loading models, only one of the dense gradation asphalt mixture AC-13C that were composed of SBS modified asphalt was applied in the laboratory tests. The strength of used asphalt mixture with various loading modes and various loading rates were investigated. The whole curves of strength with the variation of loading rates under three loading modes were obtained, and based on these, the strength mechanism with loading rates under three loading modes was explained preliminarily from the mechanical view. It is noteworthy that the achieved strength and loading rate of asphalt mixtures were treated using a standardized method. According to the mentioned research works, the main conclusions of this study are as follows:

1. Loading rate had a significant effect on the strength of the asphalt mixtures. The pattern of variation of the direct tensile strength, indirect tensile strength, and unconfined compressive strength vary with loading rate are shown in the equations *R_D_* = 2.15852*v*^0.21307^, *R*^2^ = 0.952; *R_T_* = 2.24289*v*^0.22571^, *R*^2^ = 0.957; and *R_C_* = 9.81584*v*^0.22107^, *R*^2^ = 0.992, respectively.

2. Under the same laboratory conditions, the strength of the asphalt mixture was affected by the loading mode. Among the three loading modes, the value of the unconfined compressive strength was the largest, followed by the indirect tensile strength. The strength difference under different loading modes was explained by the structural composition of the asphalt mixture. 

3. Unified strength models of asphalt mixtures under different loading modes could be depicted as *S/S*_0_ = 1.01266(*v/v*_0_)^0.21969^, *R*^2^ = 0.988. The proposed model could be applied to remove the uncertainties of strength parameters under different loading modes. Through the unified strength model, as long as the strength value under one loading mode was achieved, the strength values under the other two modes can be obtained, which greatly improved the efficiency of the laboratory test.

This study’s main aim was to report the methodology to develop a unified strength model based on one special materials. However, in order to further confirm the reliability and effectiveness of the proposed model, more types of materials should be applied in later works. 

## Figures and Tables

**Table 1:** Test results of SBS (I-D) modified asphalt.

Test Projects		Test Standard: JTG F40-2004 (China) [62]		
		Technical Requirements	Test Results	Test Methods
Penetration (25 °C,100 g, 5 s) (0.1 mm)		40~60	55.9	T 0604-2000
Penetration index PI		≥0	0.533 (*R*^2^ = 0.997)	T 0604-2000
Ductility (5 cm/min, 5 °C) (cm)		≥20	35.1	T 0605-1993
Softening point (Ring ball) (°C)		≥60	70.5	T 0606-2000
135℃ dynamic viscosity (Pa s)		≤3	2.36	T 0615-2000
Flash point (°C)		≥230	264	T 0611-1993
Solubility (%)		99	99.8	T 0607-1993
Density (15 °C)		—	1.03	T 0603-1993
Rolling Thin Film Oven Test (RTFOT) (163 °C, 85 min)	Mass loss (%)	≤±1.0	0.22	T 0609-1993
	Residual penetration ratio (25 °C) (%)	≥65	75.1	T 0604-2000
	Residual ductility(5 °C) (cm)	≥15	23.2	T 0605-1993

**Table 2:** Densities of limestone aggregate.

Sizes of Sieve (mm)	Apparent Density (g/cm^3^)	Bulk Density (g/cm^3^)	Skin Drying Density (g/cm^3^)	Water Absorption (%)
16–13.2	2.671	2.577	2.611	1.32
13.2–9.5	2.673	2.569	2.608	1.53
9.5–4.75	2.661	2.572	2.607	1.35
4.75–2.36	2.649	-	-	-
2.36–1.18	2.642			
1.18–0.6	2.606			
0.6–0.3	2.592			
0.3–0.15	2.586			
0.15–0.075	2.615			

**Table 3:** Properties of aggregate.

Test Item	Technical Requirements	Test Results	Test Methods
Crushed stone value (%)	≤26	17.9	T 0316-2005
Apparent relative density (g/cm^3^)	≥2.6	2.71	T 0321-2005
Content of flat and elongated particles in coarse aggregate (%)	≤15	9	T 0312-2005
Content of SiO_2_ (%)	/	1.81	/

**Table 4:** Results of Marshall test at the optimal asphalt-aggregate ratio.

Asphalt Aggregate Ratio (%)	Bulk Specific Gravity (g cm^−3^)	Volume of Air Voids VV (%)	Voids Filled with Asphalt VFA (%)	Marshall Stability (kN)	Flow Value (0.1 mm)
5.2	2.44	4.51	67.20	12.71	27.89
/	/	3–5	65–75	>8	20–40

**Table 5:** Test results of direct tensile strength of asphalt mixture.

Number	Loading Rate *v* (MPa/s)	Section Area of Specimen *A* (mm^2^)	Failure Loading *F* (kN)	Strength *R_D_* (MPa)	Average Value of Strength *R_D_* (MPa)	Coefficient of Variation
1	5	2631.1	7.317	2.781	2.95	0.050
2		2596.8	8.159	3.142		
3		2581.9	7.557	2.927		
4	10	2560.2	9.398	3.671	3.487	0.055
5		2588.9	9.235	3.567		
6		2611.3	8.416	3.223		
7	20	2621.5	10.772	4.109	4.158	0.054
8		2594.5	11.561	4.456		
9		2617.7	10.233	3.909		
10	30	2600.1	11.586	4.456	4.552	0.025
11		2597.3	12.236	4.711		
12		2559.6	11.490	4.489		
13	40	2579	12.887	4.997	4.821	0.039
14		2599.8	11.858	4.561		
15		2630.1	12.901	4.905		
16	50	2671	13.040	4.882	5.012	0.019
17		2599.1	13.276	5.108		
18		2611.8	13.179	5.046		
19	60	2567.7	13.080	5.094	5.13	0.013
20		2598.1	13.567	5.222		
21		2666.3	13.529	5.074		
22	70	2621.5	13.608	5.191	5.197	0.007
23		2617.3	13.723	5.243		
24		2613.5	13.478	5.157		

**Table 6:** Indirect tensile strength test of asphalt mixture.

Number	Loading Rate *v* (MPa/s)	Section Area of Specimen *A* (mm^2^)	Failure Loading *F* (kN)	Strength *R_D_* (MPa)	Average Value of Strength *R_D_* (MPa)	Coefficient of Variation
1	5	58.5	28.948	3.111	3.258	0.044
2		60.4	33.164	3.452		
3		60.5	30.900	3.211		
4	10	60.1	34.997	3.661	3.704	0.022
5		58.9	35.778	3.819		
6		59.1	34.142	3.632		
7	20	59.3	40.709	4.316	4.41	0.041
8		59.6	40.299	4.251		
9		58.8	43.611	4.663		
10	30	59.2	46.808	4.971	4.837	0.026
11		59.7	44.307	4.666		
12		59.6	46.205	4.874		
13	40	59	47.542	5.066	5.185	0.020
14		59.8	50.621	5.322		
15		60.1	49.393	5.167		
16	50	59.7	53.129	5.595	5.487	0.037
17		61.2	55.106	5.661		
18		59.6	49.343	5.205		
19	60	60.5	55.592	5.777	5.658	0.035
20		61.4	56.810	5.817		
21		60.1	51.430	5.38		
22	70	61.7	57.814	5.891	5.784	0.029
23		60.3	53.241	5.551		
24		60.8	57.154	5.91		

**Table 7:** Test results of unconfined compressive strength of asphalt mixture.

Number	Loading Rate *v* (MPa/s)	Section Area of Specimen *A* (mm^2^)	Failure Loading *F* (kN)	Strength *R_D_* (MPa)	Average Value of Strength *R_D_* (MPa)	Coefficient of Variation
1	0.02	34.862	34.862	4.441	4.134	0.057
2		32.169	32.169	4.098		
3		30.325	30.325	3.863		
4	0.05	38.473	38.473	4.901	5.062	0.039
5		38.795	38.795	4.942		
6		41.943	41.943	5.343		
7	0.1	47.249	47.249	6.019	5.901	0.025
8		46.998	46.998	5.987		
9		44.721	44.721	5.697		
10	0.5	63.773	63.773	8.124	8.421	0.025
11		66.851	66.851	8.516		
12		67.691	67.691	8.623		
13	1	78.429	78.429	9.991	9.816	0.013
14		76.255	76.255	9.714		
15		76.483	76.483	9.743		
16	2	87.064	87.064	11.091	11.441	0.022
17		90.636	90.636	11.546		
18		91.735	91.735	11.686		

**Table 8:** Cohesive force and internal friction angles at different loading velocities.

Loading Rate (MPa/s)	Unconfined Compressive Strength (MPa)	Direct Tensile Strength (MPa)	Cohesive Force (MPa)	Internal Friction Angle (°)
5	14.01	2.95	3.214	40.702
10	16.33	3.487	3.773	40.397
20	19.035	4.158	4.448	39.900
30	20.82	4.552	4.868	39.880
40	22.187	4.821	5.171	40.015
50	23.309	5.012	5.404	40.245
60	24.267	5.13	5.579	40.616
70	25.109	5.197	5.712	41.074

**Table 9:** Strength values of different loading rates under various loading modes.

Loading Rates *v* (MPa/s)	Direct Tensile Strength *R_D_* (MPa)	Indirect Tensile Strength *R_T_* (MPa)	Unconfined Compressive Strength *R_C_* (MPa)
5	2.95	3.258	14.01
10	3.487	3.704	16.33
20	4.158	4.41	19.035
30	4.552	4.837	20.82
40	4.821	5.185	22.187
50	5.012	5.487	23.309
60	5.13	5.658	24.267
70	5.197	5.784	25.109

**Table 10:** Fitting curve equations of the relation between strength and loading rate under various loading modes.

Fitting Equation	*R* = *α* × *v^β^*		
	*α*	*β*	*R* ^2^
Direct Tensile Test	2.15852	0.21307	0.952
Indirect Tensile Test	2.24289	0.22571	0.957
Unconfined Compression Test	9.81584	0.22107	0.992

**Table 11:** Relation between strength ratio and loading rate ratio.

Loading Rate Ratio *v/v_s_*	Direct Tensile Strength Ratio	Indirect Tensile Strength Ratio	Unconfined Compressive Strength Ratio
0.071	0.568	0.563	0.558
0.143	0.671	0.640	0.650
0.286	0.800	0.762	0.758
0.429	0.876	0.836	0.829
0.571	0.928	0.896	0.884
0.714	0.964	0.949	0.928
0.857	0.987	0.978	0.966
1	1	1	1
