# Correction: Xia, C.; et al. Unified Strength Model of Asphalt Mixture under Various Loading Modes. *Materials* 2019, *12*, 889

**DOI:** 10.3390/ma13235393

**Published:** 2020-11-27

**Authors:** Chengdong Xia, Songtao Lv, Lingyun You, Dong Chen, Yipeng Li, Jianlong Zheng

**Affiliations:** 1 National Engineering Laboratory of Highway Maintenance Technology, Changsha University of Science & Technology, Changsha 410004, China; xiachengdong@stu.csust.edu.cn (C.X.); 15878778765@163.com (D.C.); lypcsust@163.com (Y.L.); zjl@csust.edu.cn (J.Z.); 2 Department of Civil and Environmental Engineering, Michigan Technological University, 1400 Townsend Drive, Houghton, MI 49931, USA; liyou@mtu.edu; 3 Guangxi Communications Design Group Co., Ltd., Nanning 530000, China

## Change in Tables

The authors wish to make the following correction to this paper [1]. Due to mislabeling, replace:

**Table 6:** Indirect tensile strength test of asphalt mixture.

Number	Loading Rate *v* (MPa/s)	Section Area of Specimen *A* (mm^2^)	Failure Loading *F* (kN)	Strength *R*_D_ (MPa)	Average Value of Strength *R*_D_ (MPa)	Coefficient of Variation
1	5	58.5	28.948	3.111	3.258	0.044
2		60.4	33.164	3.452		
3		60.5	30.900	3.211		
4	10	60.1	34.997	3.661	3.704	0.022
5		58.9	35.778	3.819		
6		59.1	34.142	3.632		
7	20	59.3	40.709	4.316	4.41	0.041
8		59.6	40.299	4.251		
9		58.8	43.611	4.663		
10	30	59.2	46.808	4.971	4.837	0.026
11		59.7	44.307	4.666		
12		59.6	46.205	4.874		
13	40	59	47.542	5.066	5.185	0.020
14		59.8	50.621	5.322		
15		60.1	49.393	5.167		
16	50	59.7	53.129	5.595	5.487	0.037
17		61.2	55.106	5.661		
18		59.6	49.343	5.205		
19	60	60.5	55.592	5.777	5.658	0.035
20		61.4	56.810	5.817		
21		60.1	51.430	5.38		
22	70	61.7	57.814	5.891	5.784	0.029
23		60.3	53.241	5.551		
24		60.8	57.154	5.91		

with

**Table 6:** Indirect tensile strength test of asphalt mixture.

Number	Loading Rate *v* (MPa/s)	Height of Specimen *h* (mm)	Failure Loading *F* (kN)	Strength *R*_T_ (MPa)	Average Value of Strength *R*_T_ (MPa)	Coefficient of Variation
1	5	58.5	28.948	3.111	3.258	0.044
2		60.4	33.164	3.452		
3		60.5	30.900	3.211		
4	10	60.1	34.997	3.661	3.704	0.022
5		58.9	35.778	3.819		
6		59.1	34.142	3.632		
7	20	59.3	40.709	4.316	4.41	0.041
8		59.6	40.299	4.251		
9		58.8	43.611	4.663		
10	30	59.2	46.808	4.971	4.837	0.026
11		59.7	44.307	4.666		
12		59.6	46.205	4.874		
13	40	59	47.542	5.066	5.185	0.020
14		59.8	50.621	5.322		
15		60.1	49.393	5.167		
16	50	59.7	53.129	5.595	5.487	0.037
17		61.2	55.106	5.661		
18		59.6	49.343	5.205		
19	60	60.5	55.592	5.777	5.658	0.035
20		61.4	56.810	5.817		
21		60.1	51.430	5.38		
22	70	61.7	57.814	5.891	5.784	0.029
23		60.3	53.241	5.551		
24		60.8	57.154	5.91		

The authors wish to make the following correction to this paper [1]. Due to mislabeling, replace:

**Table 7:** Test results of unconfined compressive strength of asphalt mixture.

Number	Loading Rate *v* (MPa/s)	Section Area of Specimen *A* (mm^2^)	Failure Loading *F* (kN)	Strength *R*_D_ (MPa)	Average Value of Strength *R*_D_ (MPa)	Coefficient of Variation
1	0.02	34.862	34.862	4.441	4.134	0.057
2		32.169	32.169	4.098		
3		30.325	30.325	3.863		
4	0.05	38.473	38.473	4.901	5.062	0.039
5		38.795	38.795	4.942		
6		41.943	41.943	5.343		
7	0.1	47.249	47.249	6.019	5.901	0.025
8		46.998	46.998	5.987		
9		44.721	44.721	5.697		
10	0.5	63.773	63.773	8.124	8.421	0.025
11		66.851	66.851	8.516		
12		67.691	67.691	8.623		
13	1	78.429	78.429	9.991	9.816	0.013
14		76.255	76.255	9.714		
15		76.483	76.483	9.743		
16	2	87.064	87.064	11.091	11.441	0.022
17		90.636	90.636	11.546		
18		91.735	91.735	11.686		

with

**Table 7:** Test results of unconfined compressive strength of asphalt mixture.

Number	Loading Rate *v* (MPa/s)	Section Area of Specimen *A* (mm^2^)	Failure Loading *F* (kN)	Strength *R*_C_ (MPa)	Average Value of Strength *R*_C_ (MPa)	Coefficient of Variation
1	0.02	7850.03	34.862	4.441	4.134	0.057
2		7849.93	32.169	4.098		
3		7850.12	30.325	3.863		
4	0.05	7850.03	38.473	4.901	5.062	0.039
5		7850.06	38.795	4.942		
6		7850.08	41.943	5.343		
7	0.1	7849.98	47.249	6.019	5.901	0.025
8		7850.01	46.998	5.987		
9		7849.92	44.721	5.697		
10	0.5	7849.95	63.773	8.124	8.421	0.025
11		7850.05	66.851	8.516		
12		7850.05	67.691	8.623		
13	1	7849.96	78.429	9.991	9.816	0.013
14		7850.01	76.255	9.714		
15		7850.05	76.483	9.743		
16	2	7849.97	87.064	11.091	11.441	0.022
17		7849.99	90.636	11.546		
18		7849.99	91.735	11.686		

The authors would like to apologize for any inconvenience caused to the readers by these changes.
